# Fine structure of the intercalated disc and cardiac junctions in the black widow spider *Latrodectus mactans*

**DOI:** 10.1186/s42649-020-00040-9

**Published:** 2020-09-25

**Authors:** Yan Sun, Seung-Min Lee, Bon-Jin Ku, Myung-Jin Moon

**Affiliations:** Department of Biological Sciences, Dankook University, 119 Dandae-ro, Cheonan, 31116 South Korea

**Keywords:** Fine structure, Cardiac muscle, Intercalated disc, Cardiac junction, Spider, *Latrodectus mactans*

## Abstract

Arthropods have an open circulatory system with a simple tubular heart, so it has been estimated that the contractile pumping structure of the cardiac muscle will be less efficient than that of vertebrates. Nevertheless, certain arthropods are known to have far superior properties and characteristics than vertebrates, so we investigated the fine structural features of intercalated discs and cardiac junctions of cardiac muscle cells in the black widow spider *Latrodectus mactans*. Characteristically, the spider cardiac muscle has typical striated features and represents a functional syncytium that supports multiple connections to adjacent cells by intercalated discs. Histologically, the boundary lamina of each sarcolemma connects to the basement membrane to form an elastic sheath, and the extracellular matrix allows the cells to be anchored to other tissues. Since the intercalated disc is also part of sarcolemma, it contains gap junctions for depolarization and desmosomes that keep the fibers together during cardiac muscle contraction. Furthermore, fascia adherens and macula adherens (desmosomes) were also identified as cell junctions in both sarcolemma and intercalated discs. To enable the coordinated heartbeat of the cardiac muscle, the muscle fibers have neuronal innervations by multiple axons from the motor ganglion.

## Introduction

Cardiac muscle is an involuntary striated muscle that is found in the myocardium. The muscle has many similar properties with skeletal muscle, but there are some important differences. Each cardiac muscle cell is a single cell not multi-nucleated like skeletal muscle (Craig and Woodhead 2006). Therefore, the unique junctions called intercalated discs link the cells together and define their borders (Stenger and Spiro 1961; Sommer and Waugh 1978).

In particular, the primary role of the intercalated disc is to hold the adjacent cells together by providing sites of strong adhesion (Goossens et al. 2007). In addition, these structures are highly specialized and enable the coordinated function of the heart cells to allow the heart to beat (Franke et al. 2006). Thus, intercalated disc allows impulses to travel rapidly between adjacent cells so the heart can beat almost as a single unit, rather than beating cells separately (Forbes and Sperelakis 1985).

Previous reports have shown that the membranes of the intercalated discs establish specific associations with a variety of intracellular and extracellular structures (Stenger and Spiro 1961; Sommer and Waugh 1978; Forbes and Sperelakis 1985) as well as with numerous types of proteins (Forbes and Sperelakis 1985; Bennett 2018). In particular, the intercalated disc component which attract the interest is the gap junction (Severs 1989; Franke et al. 2006; Ehler 2016). To understand the architecture and function of the gap junction, the application of a range of microscopical approaches has been achieved to reveal whether the gap junction of intercalated discs is required for transfer of electrical excitation between cells (Forbes and Sperelakis 1985; Gourdie et al. 1991; Veeraraghavan et al. 2014).

The anatomy of spiders includes many characteristics shared with other arachnids, however spiders have several adaptations that distinguish them from other arachnids (Sherman 1987). In particular, the heart of spider is located in the abdomen above the intestine, and is not divided into chambers but consists of a simple tube that pumps hemolymph into the heart during diastole (Kim and Moon 2018; Sun et al. 2020). Moreover, a thin-walled pericardium completely surrounds the heart and gaseous exchange occurs through the book lungs (Foelix 2011).

Previous studies have shown that the cardiac muscle cells of spider are also striated and each cell is arranged to form a multiple connection with neighboring cells (Kim and Moon 2018). In addition, intercalated discs of spider cardiac muscle contain abundant gap junctions and two types of intercellular junctions - adherens junctions and desmosomes (Sun et al. 2020). However, it has been reported that there is a difference between the fine structural properties of spider cardiac muscle and that of vertebrates (Fawcett and McNutt 1969; Hoyle 1969). Furthermore, it has been previously suggested that the sliding filament model of muscular contraction may require some revision when applied to the hearts of the arthropod animals (Kawaguti 1963; Leyton 1971).

Among poisonous spiders, *Latrodectus* spider of the family Theridiidae is considered to be particularly harmful to human because of the neurotoxin (latrotoxin) (Maretić 1987). The latrotoxin acts on nerves, causing massive release of neurotransmitters and painful muscle contractions (Vetter and Isbister 2008). Because of its high toxicity, *Latrodectus* spiders have received both of biological and medical attentions (Timms and Gibbons 1986; Moss and Binder 1987; Isbister 2003; Peterson 2006).

Despite the interest in the properties of the poisonous spider’s heart, there is little information about the relationship between fine structure and mechanical activity, except for the recent work accomplished by Kim and Moon (2018). To begin filling this gap, this study defines the fine structure of the intercalated disc and cardiac junctions in the black widow spider *Latrodectus mactans*, which is notorious for its neurotoxic venoms that act specifically on nerve cells.

## Materials and methods

The black widow spiders, *Latrodectus mactans* Fabricius (Araneae: Theridiidae), were collected at Southern California and reared in rectangular cages at the laboratory of the Department of Biological Sciences, University of New Hampshire, NH, USA. All spiders were maintained under ambient conditions with natural lighting in wooden frames (15 × 20 × 15 cm) with glass plates front and back, and fed the spiders with a supply of insect food (larvae of mealworm beetles) and daily water.

Specimens were anesthetized with CO_2_ and dissected under light microscope in a drop of spider Ringer’s solution consisting of 160 mM NaCl, 7.5 mM KCl, 4 mM CaCl_2_, 1 mM MgCl_2_, 4 mM NaHCO_3_, 20 mM glucose, pH 7.4 (Moon and Tillinghast 2013; Moon 2018). Whole hearts from female specimens were quickly removed and fixed in a mixture of 2% paraformaldehyde and 2.5% glutaraldehyde buffered with cacodylate buffer. Post-fixation was performed with 1% osmium tetroxide in the same buffer and washed several times in cacodylate buffer following fixation.

For transmission electron microscopy examination, the tissues were dehydrated in graded concentrations of ethanol and propylene oxide, and embedded in Poly/Bed 812-Araldite mixture (Polysciences Inc., Warrington, PA, USA). Semi-thin sections, 0.5–1.0 μm thick stained with 1% toluidine blue (dissolved in 1% borax), were photographed using Zeiss Axiophot microscope (Carl Zeiss, Jena, Germany) coupled with Motic digital imaging system (Motic Instruments Inc., Richmond, Canada) to study the gross morphology of the heart (Kim and Moon 2018).

Ultrathin sections were obtained from a Ultracut II (Leica, Wetzlar, Germany) using an Ultra 45° diamond knife (Diatome, Hartfield, PA, USA), and were double stained with uranyl acetate and lead citrate. After these treatments, the sections were examined with a high resolution bio-transmission electron microscope JEM 2100 Plus TEM (JEOL, Tokyo, Japan) at the Korea Basic Science Institute (KBSI) of Daeduck headquarter. Captured graphic images were edited using the Photoshop software of the Adobe CS3 package (Adobe Systems Incorporated, San Jose, CA, USA).

## Results

**Fig. 1:**
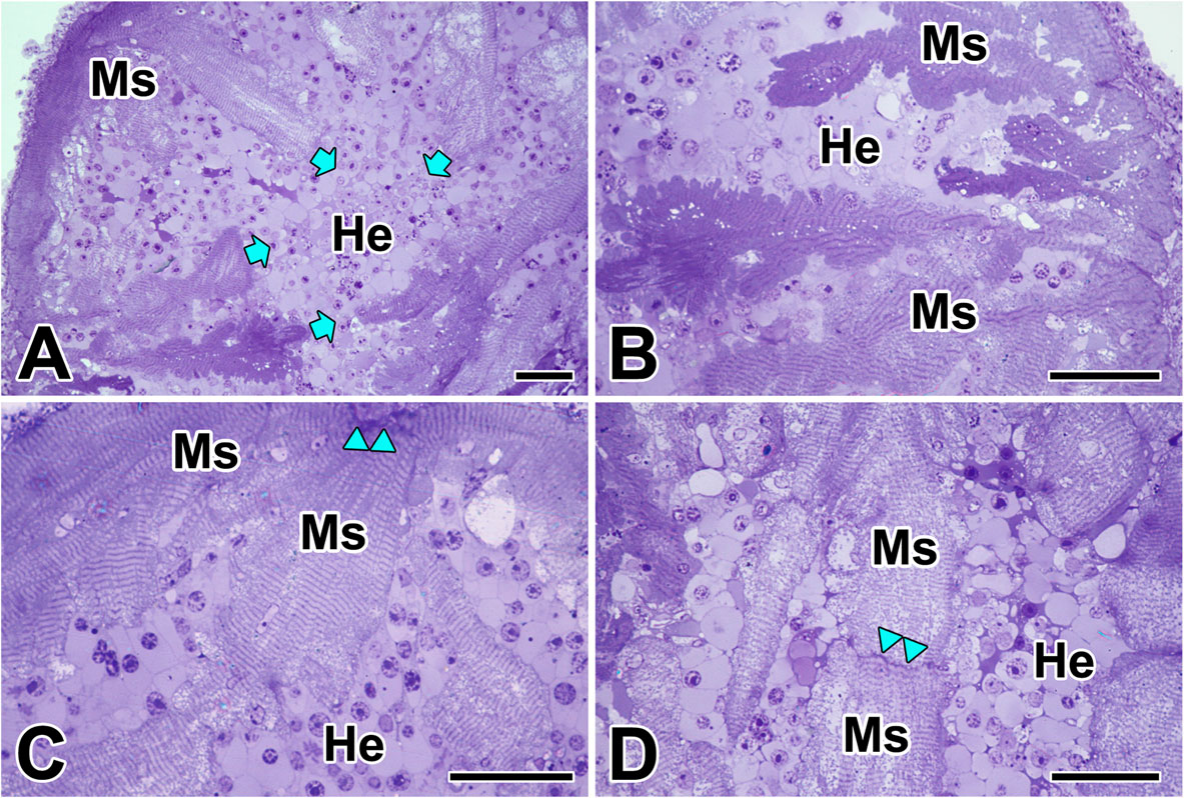
Photo micrographs of toluidine blue-stained plastic-embedded sections of the heart in the spider, *L. mactans.* **a**, **b**: Spider heart is not divided into chambers, but consists of a simple muscular tube. Arrows indicate muscle fibers (Ms) surrounding numerous hemocytes (He). **c**, **d**: Cardiac muscle fibers are striated, and tightly connected to other cells by the intercalated disc (Cd). Double arrowheads indicate the location of intercalated discs between adjacent cardiac muscle fibers. All scale bar indicates 50 μm

The heart in the black widow spider is located in the opisthosoma near the middle line of dorsal body wall. Spider’s heart is not divided into chambers, but consists of a simple muscular tube. Numerous hemocytes are accumulated at the inner surface of the myocardial layer or along the myocardial folds which stretched toward heart lumen (Fig. 1a). The heart tube is consisted of the thin outer layer of connective tissue (epicardium) and the thick muscle layer (myocardium). The myocardium in *L. mactans* has a typical fan-like spiral structure, and the muscle cells are in direct contact to the hemolymph without intima (Fig. 1b).

The muscle fibers are striated and myofibrils are densely oriented over the length of the fibers. The individual cardiac muscle cells are tightly connected to another cell by intercalated disc (Fig. 1c). Under light microscopy, intercalated discs appear as thin, typically dark-staining lines dividing adjacent cardiac muscle fibers and running perpendicular to the direction of muscle fibers (Fig. 1d).

**Fig. 2:**
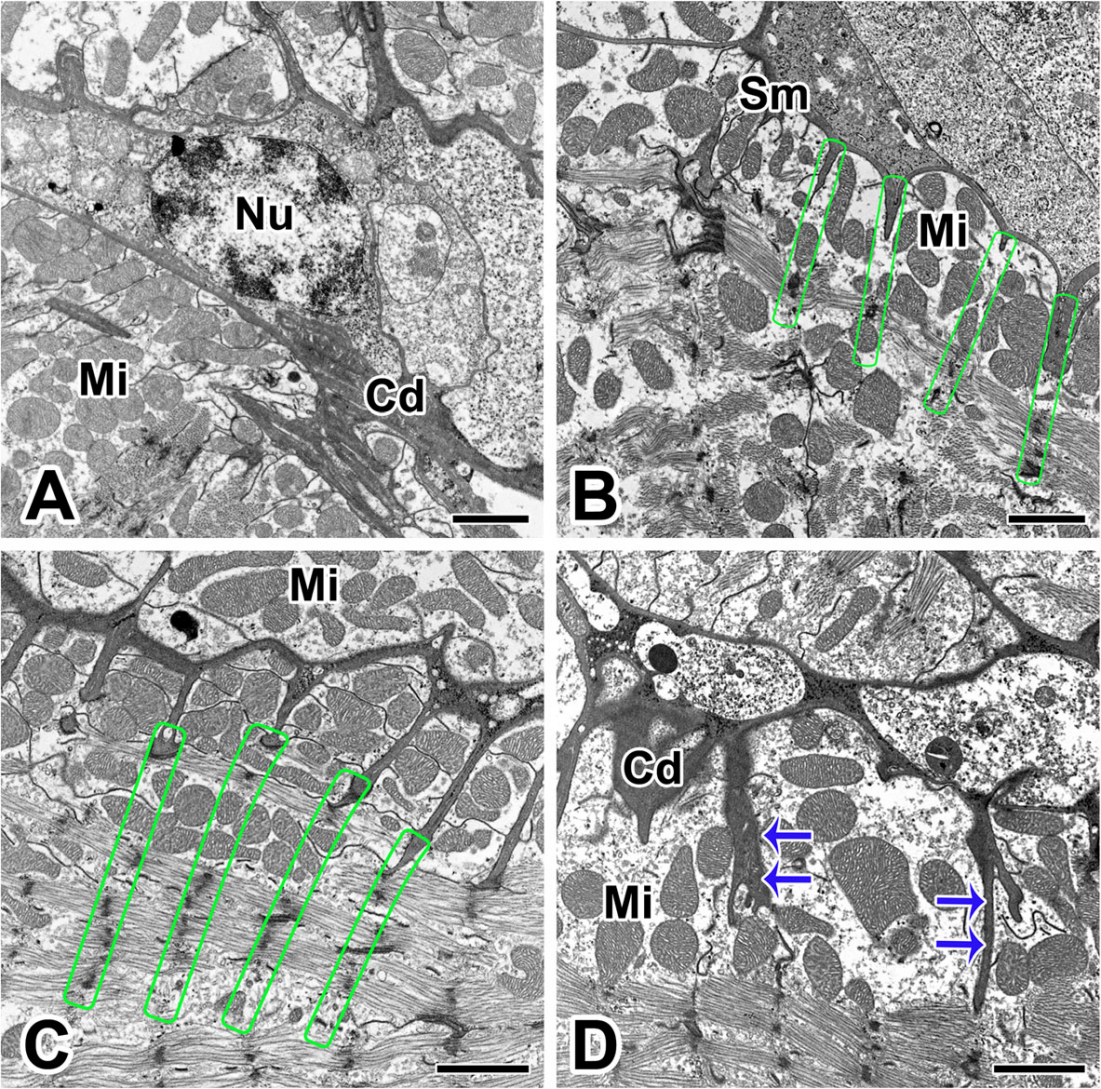
Transmission electron micrographs of the intercalated discs of the cardiac muscles in *L. mactans.* **a**: The intercalated disc (Cd) at the ends of muscle cells consist of extensive folds and intercellular junctions. **b**: Each muscle fiber contains plenty of mitochondria (Mi) for cardiac contraction. Each rectangle represents the location of Z-line of sarcomeres. **c**: The sarcolemma (Sm) of the intercalated disc shows a scalloped appearance with Z-line connections to sarcolemmal membranes (rectangles). **d**: Intercalated discs are part of the cardiac muscle sarcolemma and they contain a network of cellular junctions (arrows). Nu: nucleus. All scale bar indicates 2 μm

In cardiac muscles of the heart, connections between neighboring cells are formed by the intercalated discs. To enable the heartbeat, the intercalated disc is highly specialized and allows for coordinated function of the heart cells. First of all, the modification of the cell membrane of adjacent cardiac muscle cells is remarkable. The intercalated disc at the ends of muscle cells consist of extensive folds and intercellular junctions for mechanical and electrical connections between adjacent cells (Fig. 2a).

The plasma membrane of the muscle fiber is surrounded by a thick extracellular membrane attached to the outer surface of the plasma membrane. In particular, the sarcolemma penetrated into muscle fibers, resulting in the appearance of a scalloped border due to membrane invagination (Fig. 2b). Each muscle fiber contains plenty of mitochondria to fulfill the high demand of energy for cardiac contraction. The mitochondria between myofibrils are compactly distributed in the junctional region surrounded by the scalloped border of the sarcolemma (Fig. 2c).

The plasma membrane of cardiac muscle has a basic unit membrane which composed of two boundary lamina and a plasma membrane. The boundary lamina or external membrane is located on the outer surface of the plasma membrane. It is fairly wide with respect to the plasma membrane. There is a slight invagination on both outer and inner surfaces of the plasma membrane, representing the occurrence of micropinocytosis (Fig. 2d).

**Fig. 3:**
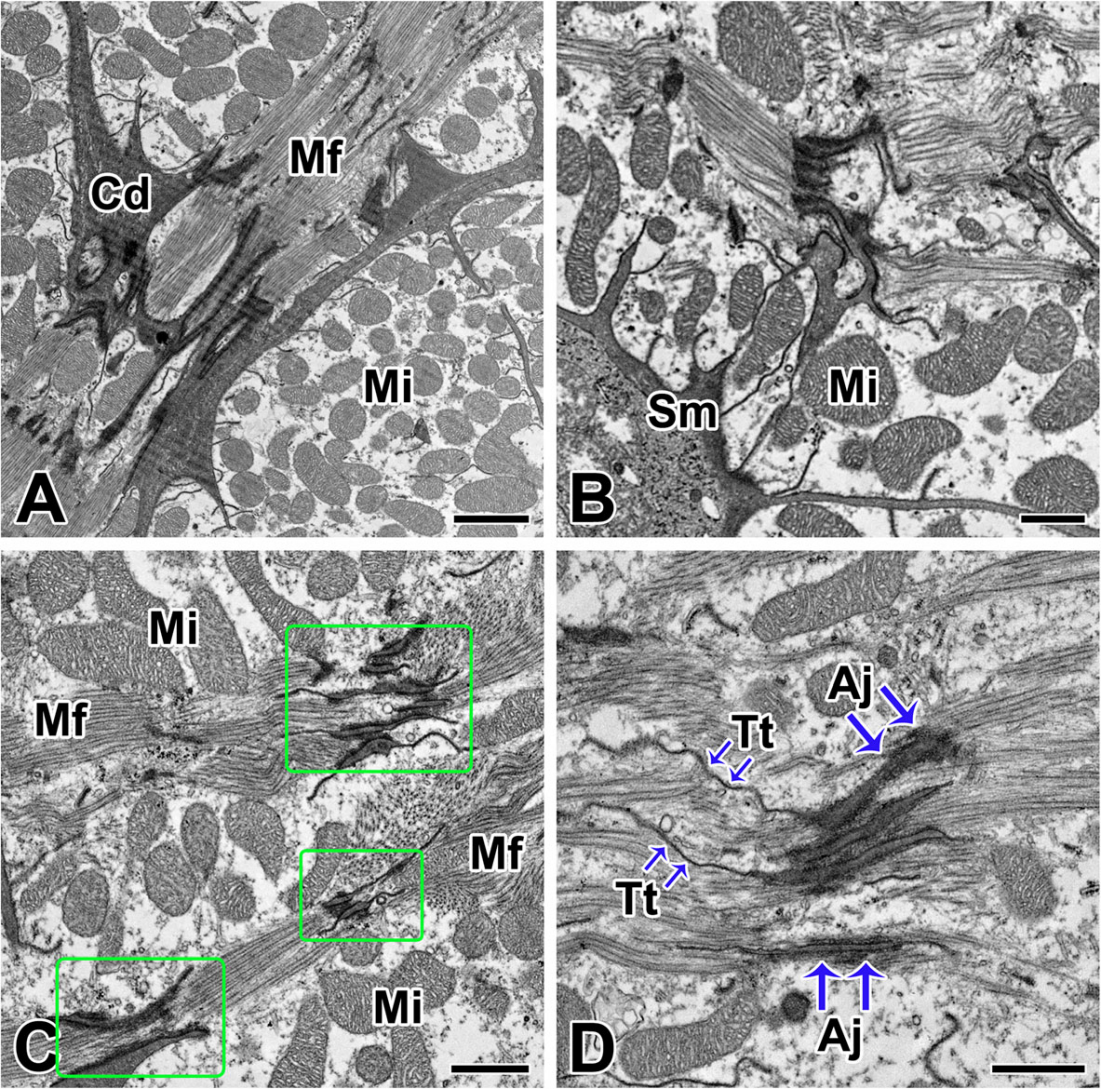
Transmission electron micrographs of the intercalated disc in the spider, *L. mactans.* **a**: Intercalated discs (Cd) run perpendicular to the direction of the myofibrils (Mf) dividing adjacent cardiac muscle cells. **b**: Well-developed mitochondria (Mi) are located very close to the intercalated disc. **c**: Intercalated discs contain gap junctions and desmosomes (rectangles) at the junctional area between muscle fibers. **d**: In cross section, isolated polygonal tubules representing the site of the gap junction, and adherens junction (Aj) appeared. Tt: T-tubule. Scale bars indicate 2 μm (A) and 1 μm (B-D), respectively

Typically, intercalated discs run perpendicular to the direction of the muscle fibers, dividing adjacent cardiac muscle cells, but the path of the intercalated disc appears more complicated under electron microscopy. In *L. mactans*, it appears to be a convoluted electron dense structure overlying the location of the obscured Z-line. In the longitudinal section, the path of intercalated disc appears to be convoluted with both the longitudinal and transverse regions (Fig. 3a). Intercalated discs are part of the cardiac muscle sarcolemma, so they also contain gap junctions for depolarization between muscle fibers and desmosomes that hold the fibers together during cardiac muscle contraction (Fig. 3b).

Well-developed mitochondria are located very close to the intercalated disc and are compactly distributed in the cytoplasm near the sarcolemma. The structure of the membrane in the intercalated disc greatly increases the surface area contact between the cells and helps hold the cells together (Fig. 3c). Along the intercellular margins of the intercalated discs, it was observed that electron dense substances connect the two inner surfaces of the interdigitated plasma membranes. In the cross section, where the interdigitation was cut transversely, isolated polygonal tubules were observed, representing that it was the site of the gap junctions of the cardiac muscle (Fig. 3d). However, clear image of the gap junction is difficult to distinguish using the ordinary transmission electron microscopic technique.

**Fig. 4:**
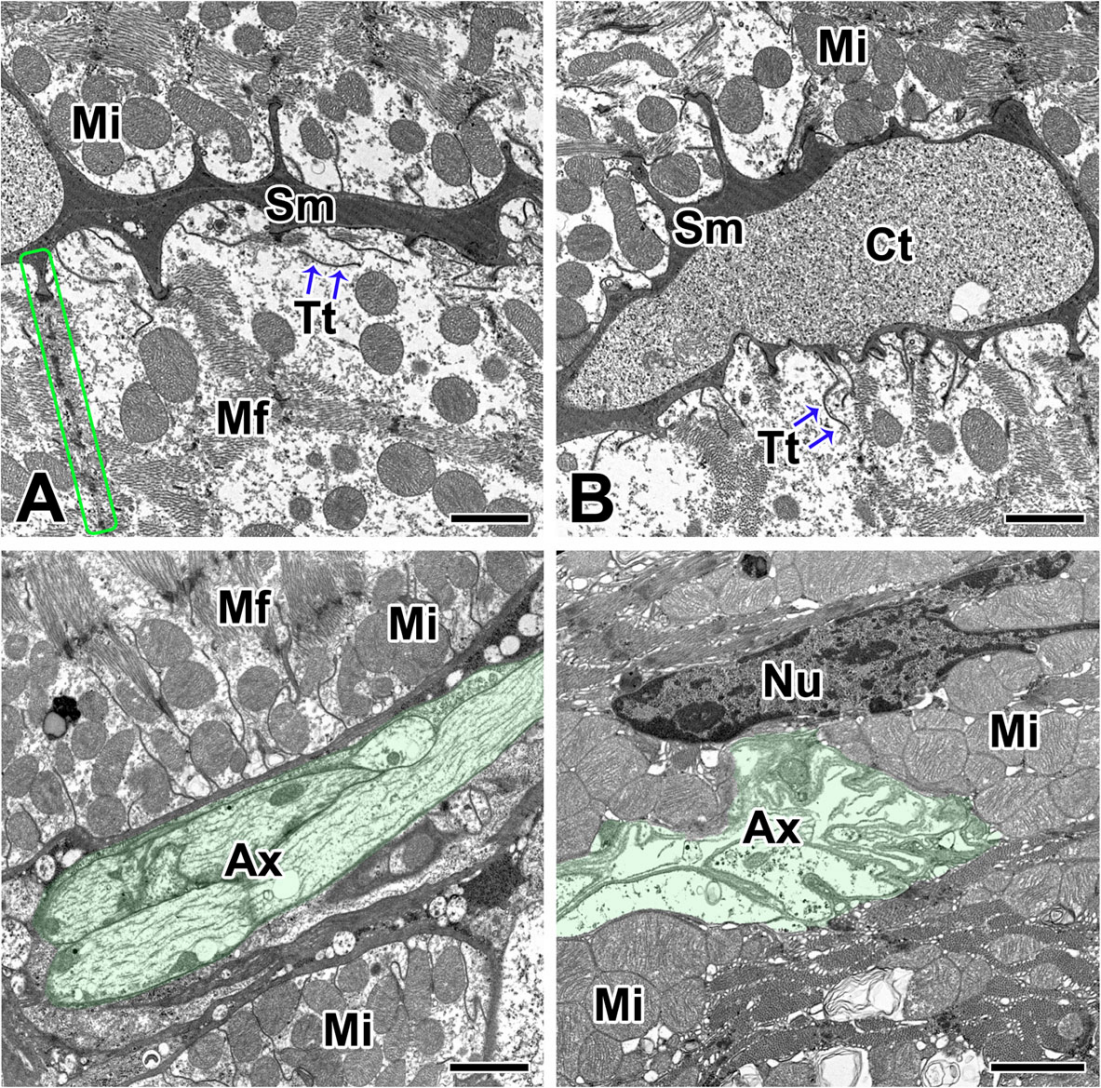
Transmission electron micrographs of the cardiac muscle cell and its neural innervation in the spider *L. mactans.* **a**, **b**: Sarcolemma (Sm) is connected with the basement membrane which surrounds connective tissues (Ct), or to other muscle cells creating a very strong fiber. **c**, **d**: Cardiac muscle fiber is innervated by neural bundles containing axons (Ax) from neurons of the cardiac ganglion. **d**: Neuronal axons are connected to the individual muscle fibers at the neuromuscular junction. Mf: myofbril, Mi: mitochondria, Nu: nucleus, Tt: T-tubules. All scale bar indicates 2 μm

Cardiac muscle fibers are surrounded by a specialized cell membrane so called sarcolemma. In cardiac muscle fibers of *L. mactans*, the sarcolemma is similar to a typical plasma membrane, but is very large compared to cell membranes (Fig. 4a). This sarcoplasmic membrane is connected with the basement membrane which surrounds connective tissues, or to other muscle cells creating a very strong fiber which can contract together. They are a delicate elastic sheath covering every muscle fiber contains an extracellular matrix which allows the cell to anchor into the other tissues that support muscle fibers (Fig. 4b).

Each cardiac muscle fiber is innervated by neural bundles from motor neuron, and the neural bundles are composed of a number of axons characterized by the absence of glial components. Axons with small dense granules make neuromuscular junctions with the cardiac muscle fibers (Fig. 4c). In *L. mactans*, axon branches through the muscle and connects to the individual muscle fibers at the neuromuscular junction. Impulses arriving on the nerve fiber are transmitted to the sarcolemma and cause the contraction of the muscle fiber (Fig. 4d).

**Fig. 5:**
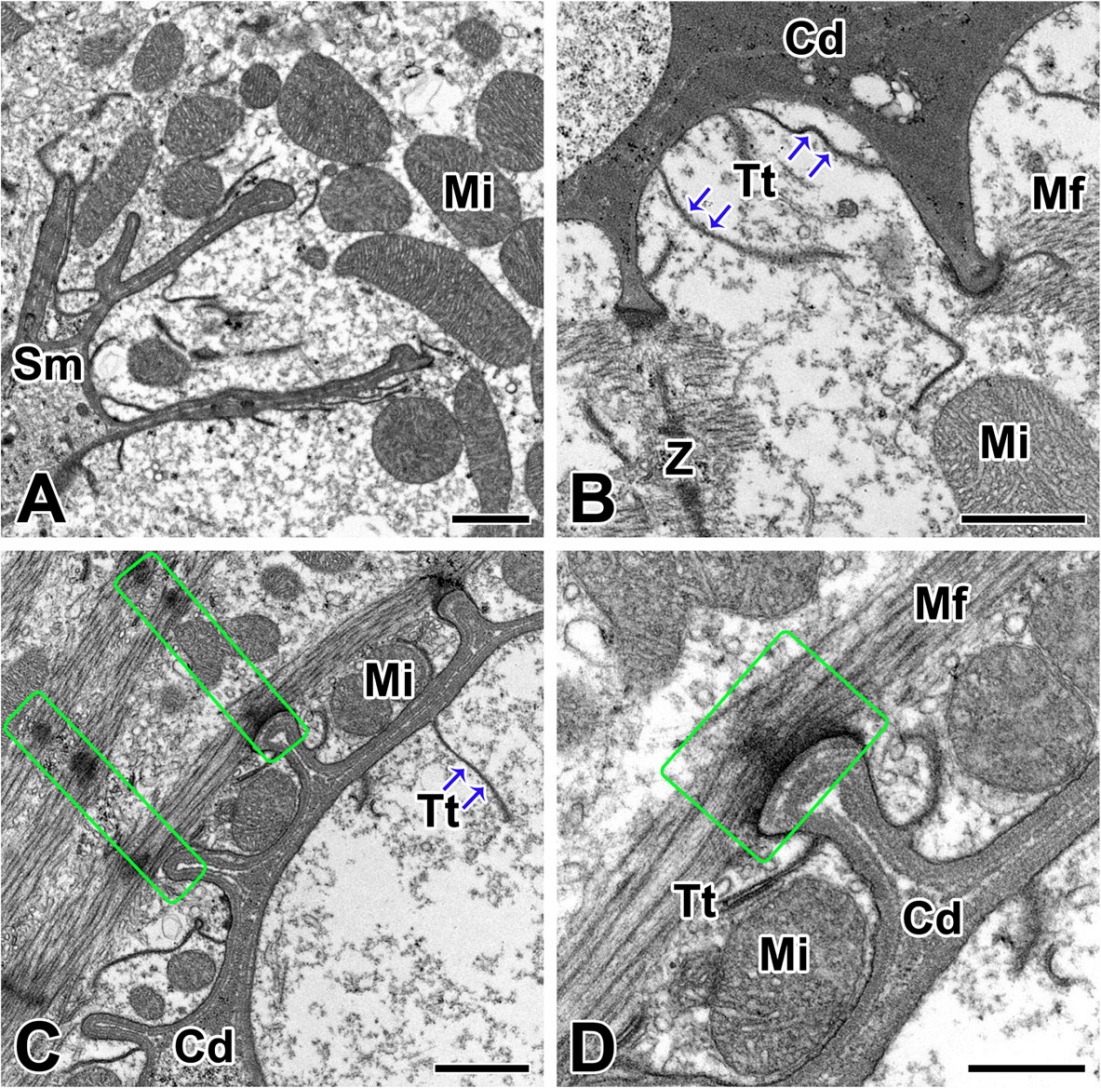
Transmission electron micrographs of the sarcolemma and its cardiac junctions in the spider *L. mactans.* **a**: Sarcolemma (Sm) of the cardiac muscle is composed of two dense membranes separated by a relatively clear space. **b**: Intercalated disc (Cd) shows deep invagination of sarcolemma to increase its surface area. **c**: Intercalated disc shows a Z-line (Z) connection to sarcolemma (rectangles) and T-tubules (Tt). **d**: Both of adherens junctions (rectangle) are observed in the sarcolemma of the intercalated disc. Mf: myofibril, Mi: motochondria. Scale bars indicate 1 μm (A-C) and 0.5 μm (D), respectively

The sarcolemma of the cardiac muscle is very large and thick compared to other cell membranes. The sarcolemma extends longitudinally between the myofibrils and crosses the myofibrils transversely. These consist of two dense membranes separated by a relatively clear space surrounded by the inner surface of an irregular band of electron dense materials (Fig. 5a). The cardiac junction is an undulating double membrane separating adjacent cardiac muscle fibers, and appears to cross the myofibrils in the plane where the Z-lines are visible. The sarcolemma at the junctional area has deep invagination to increase its surface area, and T-tubules are extended through the cell by tubular invagination (Fig. 5b).

Among the known cellular junctions of cardiac muscles, two types are evident in the cardiac muscle fibers of *L. mactans* - the fascia adherens and the macula adherens (desmosomes). The fascia adherens is a broad intercellular junction both of sarcolemma and intercalated disc of cardiac muscle fibers that anchors actin filaments. This type of junction is found at the peripheral region of cellular attachment where actin filaments of the I-band insert and terminate. In particular, a macula adherens is developed in areas of the scalloped border of the sarcolemma between the sites of filament insertion (Fig. 5c, d).

## Discussion

The open circulatory system of arthropods consists a dorsal heart and arteries, but there are no veins to bring the blood back to the heart. So, spider heart is simply an aorta or other blood vessel, and the hemolymph is pulsed throughout the body by muscle contractions (Wirkner 2010; Wirkner et al. 2013). Nevertheless, basic principles and structural organization of cardiac muscle cells are quite similar with more complex system of vertebrates (Kim and Moon 2018; Sun et al. 2020).

Basically, spider heart is a simple muscular tube that composed of two layers of heart wall, the internal myocardium and the external epicardium. The myocardium is the muscular tissue responsible for the contraction of the heart, and intercalated discs are present in this layer (Sommer and Waugh 1978). The myocardium in *L. mactans* showed as a thick muscle layer which are in direct contact to the hemolymph without intima.

In vertebrates, tunicates, and some molluscs, the heart beat is initiated and regulated by specialized groups of muscle cells, myocardial conducting cells. Although they are one of specialized cardiac muscle cells, the myocardial conduction cells initiate the action potential myogenically (Gordon et al. 2000; van Weerd and Christoffels 2016). But it has been reported that some invertebrates including insects, heart contraction is initiated and regulated by external nerve (Sherman 1987). Our TEM observation clearly shows that the cardiac muscle fiber of this spider is innervated by a branch of external nerves through neuromuscular junctions. It means that this spider’s heart is not myogenically driven but neurogenically driven.

Typically, cardiac muscles are connected with neighboring cells by intercalated discs, and they are observed under light microscope as dark-staining lines running perpendicular direction of muscle fibers. In cardiac muscle of *L. mactans*, a unique characteristic is the presence of dark-staining transverse lines that cross the cardiac cells at irregular intervals. They occur at the Z line of the sarcomere and can be visualized easily when observing a longitudinal section of the tissue. The intercalated disc provides the electrochemical and mechanical connection between neighboring cardiac muscle cells (Bennett 2018). Since the intercalated discs were considered to be a kind of intracellular structure, there were general agreements that cardiac muscle is a single functional unit called a syncytium (Dewey 1969). This is because myocardial tissue is composed of many branched cells that are joined end to end by intercalated discs (Paniagua et al. 1996).

Our electron microscopic observation also reveals that the intercalated disc is consist of the plasma membrane of adjacent cells. It has been reported that the membranes of the intercalated discs establish specific associations with a variety of intracellular and extracellular structures (Forbes and Sperelakis 1985). The intercalated discs are known to provide a scaffold for myofibrils and allow for rapid spread of contractile stimuli between cells (Gutstein et al. 2003; Goossens et al. 2007). The rapid spread of such contraction allows cardiac muscles to act as a functional syncytium.

It has been known that the skeletal muscle are consists of multinucleated muscle fibers and exhibit no intercalated discs. But, cardiac muscle consists of individual heart muscle cells connected by intercalated discs to work as a single functional organ (Franke et al. 2006). Since the intercalated discs connecting cardiac muscle cells to the syncytium, to support the rapid spread of action potentials of the cardiac tissue (Forbes and Sperelakis 1985; Gourdie et al. 1991; Veeraraghavan et al. 2014), the intercalated discs can be support synchronized contraction of the myocardium. It has been previously reported that the membranes of the intercalated discs establish specific associations with a variety of proteins and glycoproteins (Forbes and Sperelakis 1985; Bennett 2018).

In cardiac muscles of vertebrates, it has been known that three basic types of cell junction make up an intercalated disc, fascia adherens, desmosomes and gap junctions (Ehler 2016). In *L. mactans*, membrane in the intercalated disc greatly increases the surface area contact between the cells, and above three types of cellular junctions are clearly identified on the intercalated discs of cardiac muscle fibers. These three membrane junctions have their own functions and capable of coordinated contraction of spider heart. Therefore, these results strengthen the premise that contractile movement of spider heart is also controlled by the sliding filament system of muscle contraction within the sarcomere.

First, an adherens junction is defined as a cell junction whose cytoplasmic face is linked to the actin cytoskeleton (Hartsock and James 2007). Thus, adherens junction in *L. mactans* is anchoring sites for actin, and connect to the end of adjacent sarcomere of cardiac muscle fibers. In particular, the fascia adherens of spider is found as a broad intercellular junction both of sarcolemma and intercalated disc. This type of junction is found at the peripheral region of cellular attachment where actin filaments of the I-band insert and terminate. This is consistent with results reported from other vertebrates, since it has been known that the adherens junctions are composed of N-cadherin as a transmembrane component that connects to the actin filaments (Meng and Takeichi 2009; Ehler 2016).

Second, the intercalated discs are irregular transverse thickenings of the sarcolemma that contain another type of adherens junctions called macula adherens, or desmosome (Zhao et al. 2019). Desmosomes hold adjacent cardiac muscle fibers together during contraction by binding intermediate filaments (Delva et al. 2009). In *L. mactans*, a macula adherens is developed in areas of the scalloped border of the sarcolemma between the sites of filament insertion. Desmosomes are known to be composed of desmosome-intermediate filament complexes (Franke et al. 2006), which is a scaffolding of cadherin proteins, linker proteins and keratin intermediate filaments (Garrod and Martyn 2008). Thus, this macular adherens junction becomes of particular importance in intercalated discs, where the heart is exposed to increased mechanical load and needs to adapt to sustain its contractile function (Pruna and Ehler 2020).

Third, the intercalated discs also act as anchorage points for the contractile proteins, and they contain important channels called gap junctions (Forbes and Sperelakis 1985; Gourdie et al. 1991; Veeraraghavan et al. 2014). These cytoplasmic connections of adjacent cardiac muscle fibers permit the rapid spread of action potentials from one cell to another (Goodenough and Paul 2009). When gap junctions are abundant, membranes stain darker and histologists named these areas intercalated discs. Therefore, intercalated discs are gap junctions that link adjacent cardiac muscles so that electrical impulses can travel between cells and causes to contract almost simultaneously (Severs 1989). In *L. mactans*, intercalated discs are part of the cardiac muscle sarcolemma, so they also contain gap junctions for impulse conduction between muscle fibers. This is particularly true, because the gap junctions provide ion channels for intercellular communication between cardiac cells (Gutstein et al. 2003), producing depolarization of the heart muscle (Franke et al. 2006). Thus, cardiomyocytes are capable of coordinated contraction, controlled through the gap junctions of intercalated discs (Ehler 2016).

Recently, classification of cell junctions has been challenged by observations that classical desmosome proteins also identified in adherens junctions by immuno electron microscopy. Molecular studies have shown that intercalated discs consist for the most part of mixed type adherens junctions. Therefore, the terminology of ‘composite junction’ or ‘area composita’ was introduced to describe plaque-bearing cell-cell contacts at the intercalated disc (Franke et al. 2006). These represent an amalgamation of typical desmosomal and fascia adherens proteins in contrast to various epithelia. Thus adherens junctions in cardiac muscle differ from epithelial adherens junctions and desmosomes (Shimada et al. 2004; Borrmann et al. 2006).

Sarcolemma is used in electron microscopic studies to describe the unit membrane that encloses the cytoplasm of the muscle cell (McNutt 1975). In *L. mactans*, the sarcolemma of cardiac muscle cell has a basic unit membrane which composed of a plasma membrane and two boundary lamina. Previous studies have shown that the boundary lamina is a specialized surface coat which composed of fibrillar glycoprotein material (Forbes and Sperelakis 1985). The surface coat or glycocalyx covers most of the external aspect of the sarcolemma, so called as sarcolemma-glycocalyx complex (Lee 1987). The glycocalyx may be a site of calcium binding and exchange across the cell membrane in cardiac muscle (Adams and Schwartz 1980), because depolarization of the sarcolemma is associated with an influx of calcium into the cell.

Neural control of muscle systems in arachnids is poorly understood in comparison to the other large classes of arthropods such as the crustaceans and insects (Sherman 1987). It has been reported that spiders have the neurogenic heart from the findings of a cardiac ganglion on the heart (Wilson 1967), recording electrical impulses of heartbeat (Sherman and Pax 1968) and detailed histological examination of the cardiac ganglion (Bursey and Sherman 1970). The neurogenic heartbeat in lobsters and horseshoe crab generates periodic bursts of action potentials by the neural elements associated with the myocardium, however the myogenic heartbeat in molluscs and vertebrates initiates rhythmic contractions of the myocardium by the muscle cells itself in the heart (Sherman 1987).

Our transmission electron microscopic observation clearly shows that neuronal axons from cardiac ganglion extend into the myocardium. In addition, neuromuscular synapses are also present along the surface of the myocardial cells. This is consistent with the physiological evidence for multiple neuronal input to each myocardial cell (Sherman and Pax 1968; Ude and Richter 1974), which suggests that the pacemaker cells in spiders are modified neurons that are attached to the heart. This is an important difference comparing to vertebrates since heart contraction is not self or myogenically driven but neuronally driven. Although vertebrate hearts are innervated similarly by neurons from the autonomic nervous systems but these neurons act in only a modulatory function.

## Conclusion

- We investigated the fine structural features of intercalated discs and cardiac junctions of cardiac muscle cells in the black widow spider *Latrodectus mactans*.
- Spider cardiac muscle has typical striated features and represents a functional syncytium with adjacent cells by intercalated discs.
- Boundary lamina of each sarcolemma connects to the basement membrane to form an elastic sheath covering.
- Extracellular matrix allows the cells to be anchored to other tissues.
- Intercalated disc contains gap junctions and desmosomes for depolarization and muscle contraction.
- Fascia adherens and macula adherens (desmosomes) were identified in both sarcolemma and intercalated discs.
- Muscle fibers have neuronal innervations by multiple axons for coordinated heartbeat.

## Data Availability

Materials described in the manuscript, including all relevant raw data, will be freely available to any scientist wishing to use them for non-commercial purposes.

## Abbreviation

T-tubule: Transverse tubule
